# Polymersomes as Innovative, Stimuli-Responsive Platforms for Cancer Therapy

**DOI:** 10.3390/pharmaceutics16040463

**Published:** 2024-03-26

**Authors:** Irina Negut, Bogdan Bita

**Affiliations:** 1 Faculty of Physics, University of Bucharest, 077125 Magurele, Romania; negut.irina@inflpr.ro; 2 National Institute for Lasers, Plasma and Radiation Physics, 077125 Magurele, Romania

**Keywords:** multi-responsive polymersomes, targeted drug delivery, cancer therapy

## Abstract

This review addresses the urgent need for more targeted and less toxic cancer treatments by exploring the potential of multi-responsive polymersomes. These advanced nanocarriers are engineered to deliver drugs precisely to tumor sites by responding to specific stimuli such as pH, temperature, light, hypoxia, and redox conditions, thereby minimizing the side effects associated with traditional chemotherapy. We discuss the design, synthesis, and recent applications of polymersomes, emphasizing their ability to improve therapeutic outcomes through controlled drug release and targeted delivery. Moreover, we highlight the critical areas for future research, including the optimization of polymersome–biological interactions and biocompatibility, to facilitate their clinical adoption. Multi-responsive polymersomes emerge as a promising development in nanomedicine, offering a pathway to safer and more effective cancer treatments.

## 1. Introduction

The persistent nature of cancer, influenced by both environmental factors and genetic predispositions, has maintained its status as a leading cause of mortality into the 21st century, as evidenced by extensive research [1]. Surgical resection is the cornerstone of therapy for malignant solid tumors, often complemented by neoadjuvant treatments like chemotherapy, radiotherapy, phototherapy, and immunotherapy to enhance outcomes. These adjunct therapies, used preoperatively, have gained prominence recently for their potential to improve surgical success and patient prognosis [2]. However, the lack of specificity inherent to most chemotherapeutic agents results in the destruction of tumor cells alongside significant toxic side effects on healthy tissues, which substantially restrict their clinical utility [3].

The development of stimulus-triggered drug carrier systems, which integrate stimulus-responsive components with the therapeutic agents, represents a strategic innovation aimed at achieving precise and timely drug delivery [4,5,6]. This approach is responsive to both internal (e.g., pH, temperature, enzymes) and external (e.g., light, magnetic field) stimuli, thereby enhancing drug bioavailability and minimizing the adverse effects associated with chemotherapy [5,6]. Given the distinct microenvironmental differences between diseased sites and normal tissues, single stimulus-responsive drug delivery systems fall short of addressing the nuanced requirements of contemporary drug therapy [7].

The incorporation of materials responsive to multiple stimuli into the drug delivery paradigm facilitates adaptation to the complex milieu of the lesion site and enables controlled drug release [8,9].

Nanotechnology is a field of research and innovation focused on creating materials, devices, and systems through manipulating matter at the nanoscale, typically between 1 to 100 nanometers [10]. At this scale, materials exhibit unique physical, chemical, and biological properties not seen in their larger-scale counterparts, enabling novel applications across various disciplines including medicine, electronics, energy, and environmental science [10]. In (nano)medicine, particularly, it paves the way for precise targeting of cancer cells, enhanced drug solubility, and extended circulation time and allows for controlled drug release, ensuring sustained treatment efficacy [11,12]. The main nanotechnological tools, nanoparticles (NPs), can be engineered for multi-functional purposes, combining therapeutic and diagnostic capabilities in a single platform [13].

Polymeric nanoparticles and polymersomes represent two distinct approaches in nanotechnology for drug delivery and material science applications. Polymeric NPs can be designed with a wide variety of structures, including spheres or capsules [12,14], which encapsulate active ingredients for controlled release. Polymersomes, composed of amphiphilic block copolymers, are unique hollow nanospheres more closely resembling cellular membranes, characterized by bilayer membranes comprising hydrophobic segments and hydrophilic crowns [15]. These structures enable them to encase water-soluble substances within their internal cavity and hold hydrophobic compounds within their bilayer membranes [15]. A key advantage of polymersomes, considered an alternative to liposomes, is their enhanced chemical and mechanical stability, attributed to the higher molecular weights of their building blocks and the thicker vesicular membranes they possess [16]. However, this increased robustness comes with a downside: reduced permeability and fluidity, potentially impacting the effective release of their cargo [17]. They exhibit robust physical and chemical properties, high drug-loading capacities, excellent colloidal stability, and significant biocompatibility [17,18]. Crucially, they can encapsulate a wide range of therapeutic agents, both hydrophilic and hydrophobic, due to their unique structure comprising a hydrophobic bilayer and an aqueous core [18]. This versatility allows for targeted delivery of drugs to cancer cells, potentially enhancing treatment efficacy while minimizing damage to healthy cells.

The functionalization of polymersomes with specific ligands that target receptors overexpressed on tumor cells significantly enhances the uptake of anticancer agents [19,20,21]. This targeted approach allows polymersomes to be internalized more efficiently by cancer cells compared to healthy cells, and they exhibit lower toxicity even at higher concentrations [20].

When administered systemically, polymersomes leverage the enhanced permeation and retention (EPR) effect to accumulate passively at tumor sites, facilitating localized drug release directly at the target area [20].

Polymersomes leverage the EPR effect for tumor targeting but face challenges such as heterogeneous tumor vasculature, variable patient responses, and drug release issues. Strategies to enhance their effectiveness include surface functionalization and tuning polymersome properties for better tumor penetration and patient-specific treatments [20].

Moreover, the incorporation of stimuli-sensitive components into polymersomes allows for controlled drug release. This responsiveness to external stimuli (such as changes in pH, temperature, or light) ensures that the drug release is more precise, occurring specifically in the tumor microenvironment [22]. Due to these advantageous properties, polymersomes have garnered considerable interest in various biomedical fields. They are not only prominent in drug delivery systems [23] but also play critical roles in gene therapy [24] and diagnostic applications [25]. Their versatility and efficacy in targeting and treatment make them promising tools in the ongoing advancement of medical technologies.

### Statement of Significance

This review paper explores the state of the art in polymersome drug delivery technology in a unique way, going beyond basic principles to reveal the latest developments and unrealized possibilities in this field. In contrast to previous research, our manuscript explores the cutting-edge synthesis techniques, unique materials, and stimuli-responsive uses of polymersomes that have surfaced recently. We specifically highlight their significance in resolving long-standing issues with targeted therapy and controlled release mechanisms. Additionally, by examining the challenges and opportunities that lie in the way of practical application, this paper highlights the directions that polymersome research will go in the future, including the development of smart polymersomes and how they relate to customized treatment.

We seek to stimulate interdisciplinary collaboration, generate new research questions, and expedite the development of polymersome-based therapeutics by highlighting the future directions of polymersome research, including the emergence of smart polymersomes and their intersection with personalized medicine. Our contribution is of great significance to researchers, physicians, and policymakers alike, since it not only enhances the scholarly discourse but also emphasizes the transformative potential of polymersomes in changing drug delivery systems.

## 2. General Aspects on Designing and Fabricating Polymersomes

### 2.1. Choice of Polymer Type

**Figure 1:**
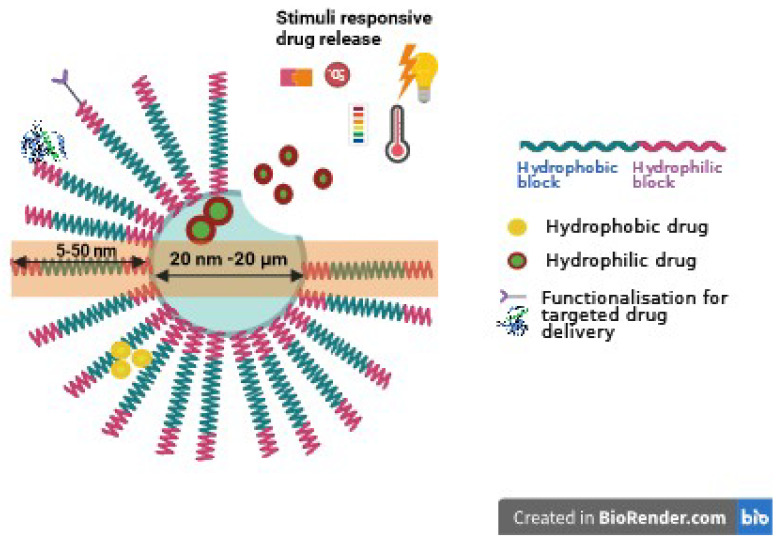
Schematic representation of a polymersome, containing hydrophilic and hydrophobic drugs loaded within its core and membranes. The surfaces of polymersomes can be further modified with selective targeting moieties. Image created with Biorender.com (accessed on 4 March 2024).

The typical structure of polymersomes, as shown in Figure 1, reveals that the core section presents hydrophilic molecules, while the hydrophobic bilayer is engineered to load hydrophobic molecules. This dual capability makes polymersomes versatile for carrying/delivering a range of therapeutic agents, enhancing their application in targeted drug delivery systems.

**Figure 2:**
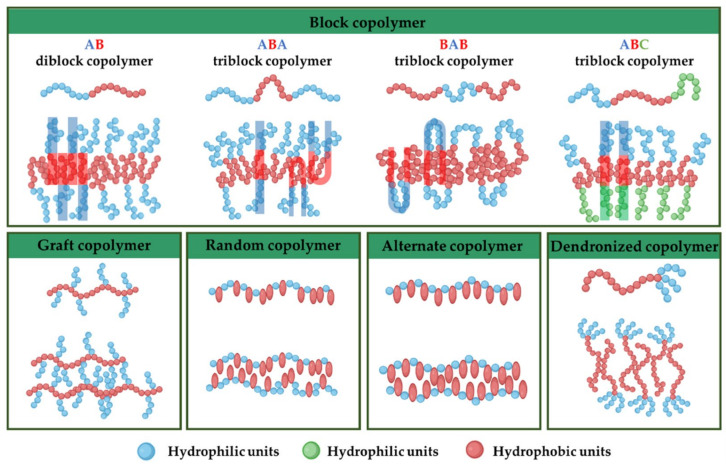
Illustration of various amphiphilic copolymers and their anticipated structural arrangement within the bilayer membrane of polymersomes [27].

Before starting the assembly process, selecting the right block copolymer is crucial because it significantly influences various attributes of the final polymersome, such as membrane thickness, stability, and biological interaction within the body [26,27]. Amphiphilic copolymers, due to their unique ability to self-assemble into a variety of nanostructures in aqueous environments, are classified based on their architectural configuration (Figure 2): (i) *block copolymers*: composed of two or more homopolymer subunits connected in a linear sequence. These subunits typically exhibit contrasting hydrophilic and hydrophobic properties, allowing for the formation of distinct domains within a given structure [27,28]; (ii) *graft copolymers* are characterized by a backbone of one type of monomer to which side chains of another monomer are attached. This architecture allows for the design of polymers with specific, localized functionalities [27,29]; (iii) *random copolymers* are made up of two or more types of monomers distributed randomly along the polymer chain. This randomness affects the copolymer’s solubility, thermal properties, and ability to form micelles [27,30]; (iv) *alternate copolymers* consist of two types of monomers arranged alternately along the polymer chain. This regular pattern can influence the physical properties and self-assembly behavior of the copolymer [27]; (v) *dendronized copolymers* have a central backbone with densely branched structures known as dendrons. These polymers exhibit unique properties due to the presence of multiple branching layers, which can enhance solubility and interaction with other molecules [27,31]; (vi) *gradient copolymers* present a gradual change in the composition or type of monomers along the polymer chain, rather than a sharp transition. This gradient can affect the polymer’s phase behavior and compatibility with different substances [27,32].

There exists a broad array of polymers that can be used to create block copolymers, offering nearly limitless possibilities. The preferred polymers for the hydrophobic segment include poly(caprolactone) (PCL) [33,34], poly(acrylates) [35], poly(methacrylates) with different substituents, and poly(lactic acid) (PLA) [36]. In contrast, the hydrophilic segments typically involve poly(ethylene glycol) (PEG) [37,38] and poly(amino acids) [39]. PEG, in particular, is frequently used as the hydrophilic component in di- and triblock copolymers due to its ability to prevent plasma protein adsorption, thereby enhancing the stability of the carrier in plasma [40]. Its neutral and hydrophilic nature also renders the polymersomes less detectable to dendritic and phagocytic cells [40].

The methodologies for synthesizing these components or the final block copolymers vary, ranging from ring-opening polymerization to reversible deactivation radical polymerizations [41]. Not limited to diblock copolymers, suitable polymer architectures may also consist of macromolecules with three or more blocks. Importantly, the structure significantly impacts the self-assembly process and the ultimate characteristics of the polymersomes [23]. Another vital factor in determining the final self-assembled shape of these block copolymers is the molecular weight ratio between the hydrophilic and hydrophobic parts. This ratio, along with the interplay of free energy and kinetic factors, dictates the final structure [42].

The structure that these self-assembled aggregates will likely adopt in a solution is predicted by the packing parameter (p = v/al) [43]. This parameter considers the volume of the hydrophobic block (v), the contact area of the head group (a), and the length of the hydrophobic block (l). According to this parameter, if p is less than 1/3, spherical structures are formed. For values between 1/3 and 1/2, cylindrical shapes emerge, and when the parameter is between 1/2 and 1, polymersomes are formed [44]. This predictive approach is fundamental in tailoring polymersomes for specific applications, such as targeted drug delivery systems, based on their desired structural characteristics [44]. However, these ratios are not exact rules; other self-assembled structures may arise, and there are often ranges of composition where different structures coexist [44].

When the concentration of block copolymers in a solution surpasses the critical aggregate concentration, these copolymers undergo self-assembly, leading to the formation of high-molecular-weight aggregates [45]. Amphiphilic block copolymers are characterized by notably slow chain exchange dynamics and possess a low critical aggregate concentration [46]. Consequently, this results in polymersomes remaining in the bloodstream for extended periods.

Polymersomes provide distinct advantages over polymeric nanoparticles due to their unique vesicular structure, allowing for the encapsulation of both hydrophilic and hydrophobic drugs [14,15]. Their bilayer configuration offers enhanced stability and controlled drug release, which can be further tuned for specific therapeutic outcomes through adjustments in membrane composition and thickness [14,15]. Additionally, polymersomes can be engineered for multi-functionality, carrying various drugs and diagnostic agents, and can be functionalized for targeted delivery [14,15]. Their design mimics biological membranes, potentially improving biocompatibility and reducing immunogenicity. Overall, the versatility and structural integrity of polymersomes make them a superior platform for drug delivery in numerous medical applications [14,15].

The EPR effect is a phenomenon which allows for the preferential accumulation of drugs and NPs in tumor tissue, as opposed to normal tissues. This effect is based on two key characteristics of tumor vasculature and the tumor microenvironment: enhanced permeability and retention [47].

Polymersomes with a bilayer thickness of ~50 nm, contributing to an overall diameter of ~100 nm, are within a size range that is relevant for the EPR effect. The EPR effect is maximally exploited by particles in the range of roughly 10 nm to 200 nm [48], where they can take advantage of the leaky tumor vasculature while avoiding rapid clearance by the kidneys (which typically clears particles < 5–6 nm) and capture by the liver and spleen (which is more efficient for particles > 200 nm) [49]. A 100 nm diameter for polymersomes means they are sufficiently large to remain in the bloodstream long enough to reach tumor sites and small enough to pass through the gaps in tumor vasculature, which can range widely but are often on the order of 100–780 nm or larger in some tumors [50]. This size also allows for the retention of polymersomes within the tumor due to the impaired lymphatic drainage. Still, the implications of the bilayer thickness itself on the EPR effect are less direct. The thickness might influence the stability and rigidity of polymersomes, potentially affecting their circulation time and interaction with the tumor environment. A thicker bilayer could confer increased stability against premature release of encapsulated drugs and protect against degradation in the bloodstream, enhancing the potential for polymersomes to reach and accumulate in the tumor tissue effectively.

One of the key aspects of polymersomes is the ability to customize the thickness of their membrane by adjusting the molecular weight of the amphiphilic polymers. Such adjustments can significantly impact their physicochemical properties, including mechanical stability and toughness [51].

### 2.2. Fabrication Techniques

The process of engineering polymersomes typically starts with dissolving an amphiphilic polymer in a selective organic solvent, forming an organic phase. This polymer-rich organic phase is then introduced to an aqueous phase. Through mixing, the polymer becomes finely dispersed in the aqueous medium, leading to the self-assembly of the amphiphilic molecules into polymersomes [52]. Various methods can be employed to fabricate polymersomes, including nanoprecipitation (also known as the solvent-exchange method), film rehydration, electroformation, and different emulsification techniques like the oil-in-water emulsion and double emulsion [53].

The design and fabrication of polymersomes have been significantly advanced by the development of controlled radical polymerization techniques such as reversible addition–fragmentation chain transfer polymerization, atom transfer radical polymerization, and nitroxide-mediated polymerization. These methods have enabled the synthesis of well-defined amphiphilic polymers with distinct features [54].

Controlled radical polymerization techniques facilitate the creation of functional polymers with specific molecular weights, precise architectures, and low polydispersity [55]. This includes the synthesis of various copolymers like block, triblock, and graft copolymers [55]. Numerous studies have demonstrated the self-assembly of these amphiphilic polymers into distinct forms such as spheres, cylinders, and polymersomes [52].

Table 1 provides a short overview of the preparation methods for polymersomes.

Creating polymersomes presents the challenge of maintaining their stability, especially in diluted conditions [18]. Polymersome formation relies on the self-assembly of amphiphilic block copolymers (ABCs) when the concentration surpasses critical levels like the critical micelle concentration (CMC) and critical aggregation concentration (CAC). The CAC is a crucial parameter which indicates the concentration at which amphiphilic molecules spontaneously form micelles or other aggregate structures in solution [62]. A lower CAC suggests that polymersomes can form stable structures at lower concentrations, enhancing their suitability for drug delivery applications by ensuring stability and uniformity in physiological conditions [16]. Polymersomes typically exhibit a lower CAC compared to polymeric NPs [16]. Regarding drug-loading capacity, polymersomes often have an advantage over polymeric NPs due to their unique architecture. Polymersomes can encapsulate a wide range of drug molecules within their aqueous interior or within the hydrophobic bilayer, potentially allowing for higher loading capacities, especially for hydrophilic drugs [16]. This is complemented by their ability to provide a controlled release, which can be finely tuned by adjusting the bilayer characteristics. In comparison, polymeric NPs, depending on their design (e.g., solid or core–shell structures), may have limitations on the solubility and compatibility of drugs within the polymer matrix, which can influence their loading capacity and release profile [63]. While polymeric NPs are versatile and can be engineered for high drug loading and targeted delivery, the vesicular nature of polymersomes generally offers a broader range of drug-loading options and more tunable release mechanisms [16,63]. The copolymer’s hydrophobic segment plays a crucial role; higher molecular weights in this segment correlate with a reduced CAC. This aspect is essential since only micelles with extremely low CACs are suitable for drug delivery, but it can limit the bioavailability of hydrophobic drugs encapsulated within these systems in vivo [64]. The aggregate shape is also determined by the ratio of hydrophilic to hydrophobic components. For example, hydrophilic volume fraction (fEO) values below 25% lead to aggregates and solid particles, 25–40% to Ps, 40–50% to hollow tubules, and above 50% to micelles [42,65].

Traditional self-assembly techniques such as solvent exchange and thin film rehydration, despite their simplicity and minimal equipment requirements, are somewhat antiquated. These methods are characterized by limited control over vesicle size, resulting in polymersomes with broad size distributions (polydispersity index, PDI > 0.2) and low encapsulation efficiencies [66]. Conversely, alternatives that are commercially available and cost-effective offer significantly improved outcomes, producing more uniform structures and frequently achieving higher encapsulation efficiencies [67]. Techniques like flash nanoprecipitation and stirred tank reactors excel by generating large volumes of polymersomes with narrow size distributions and satisfactory encapsulation efficiencies (up to 43%) [18]. Furthermore, microfluidic approaches, such as double emulsions and hydrodynamic flow focusing, are notable for producing vesicles with high precision (PDI < 0.10) and exceptionally high encapsulation efficiencies [68]. The selection of a microfluidic method should be tailored to the specific requirements of the application, considering factors such as desired encapsulation efficiency and vesicle size [68]. However, microfluidic techniques often suffer from limited throughput. Polymerization-induced self-assembly (PISA) represents a novel strategy that combines polymer synthesis with nanostructure formation, though concerns remain regarding the potential toxicity of unreacted monomers [68].

The chosen method for assembling polymersomes significantly impacts their size. For instance, electroformation and the double-emulsion technique typically yield polymersomes in the micrometer size range. In contrast, methods like film rehydration and nanoprecipitation tend to produce nanometer-sized polymersomes [52]. External factors such as sonication, freeze/thaw cycles, and extrusion through polycarbonate filters can further modify the structure of polymersomes [20].

### 2.3. Drug Loading

Polymersomes, characterized by their distinctive bilayer membranes and hollow interior structures, can concurrently accommodate both hydrophobic and hydrophilic pharmaceutical agents. This capability not only safeguards the drugs from degradation but also prolongs their presence within the circulatory system [69]. The process of loading hydrophobic substances typically involves their integration into the vesicles during the self-assembly phase, employing techniques such as nanoprecipitation, film rehydration, microfluidics, and electrostatic complexation, as well as utilizing water-in-oil-in-water (w/o/w) emulsions or diffusion methods [18,69]. To encase hydrophilic drugs, these compounds can be sequestered within the polymersome’s cavity by creating pH or ammonium salt gradients [70]. Additionally, the w/o/w emulsions approach [71] and electroporation technology [72] offer alternative strategies for the loading of hydrophilic drugs. The sequential loading of hydrophobic drugs into the vesicle membrane, followed by the introduction of hydrophilic drugs using either pH or ammonium salt gradient techniques, facilitates the coloading of both drug types [16]. The w/o/w emulsions method further supports the simultaneous incorporation of hydrophilic and hydrophobic drugs, showcasing the versatility and efficiency of polymersomes as drug delivery vehicles [16].

During the process of drug loading, the interactions between drugs and drug–polymer can significantly influence the self-assembly behavior of copolymers. A study conducted by utilizing curcumin (CUR) as a model drug revealed that increasing CUR concentrations from 0.37 to 0.75 mg/mL led to a reduction in the polymersomes’ diameter from 272 to 263 nm. Further corroborating analysis showed that CUR was predominantly localized within the shell of the polymersomes. The block copolymer employed in their investigation typically favored the formation of cylindrical micelles; however, the incorporation of CUR prompted a morphological transition to polymersomes [73]. Similarly, Ding and colleagues observed that drug complexation could trigger a micelle-to-vesicle transformation, marked by a structural shift from a random coil configuration to an α-helix conformation [74]. These findings emphasize the profound impact of drug incorporation on the structural dynamics and morphological evolution of polymer assemblies, highlighting the intricate relationship between drug loading and copolymer self-assembly processes.

While it is feasible to load therapeutic molecules during the formation of polymersomes, it is often preferable to encapsulate these molecules after fabrication. This approach helps avoid potential detrimental effects on the carrier system. Postfabrication techniques for drug encapsulation in polymersomes include extrusion, electroporation, and ultrasonication [75]. Table 2 provides a detailed overview of these various preparation methods for polymersomes. Each method accommodates specific types of drugs and desired properties of the drug delivery system, and the choice depends on the particular requirements of the therapeutic application.

A well-designed polymersome fabrication process must address multiple parameters like size, loading capacity, and dispersity. It should also consider the thermodynamics of the self-assembly process and ensure reproducibility. The encapsulation efficiency is significantly influenced by the composition of the copolymer, including its charge and the interactions between the drug and polymer. Understanding these factors is vital for optimizing the design and functionality of polymersomes, particularly for drug delivery applications.

**Figure 3:**
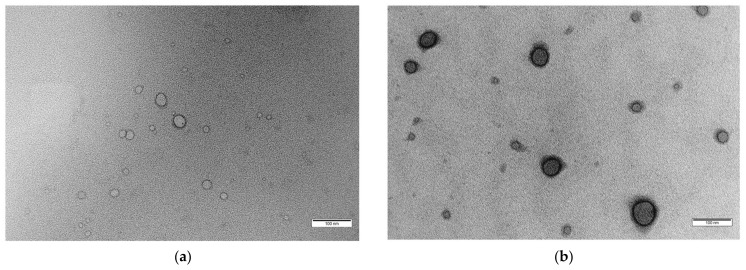
(**a**) Typical morphology of spherical vesicular structures with an inner hydrophilic core and outer hydrophobic shell of blank polymersomes formed at pH 7.4 with a polymer concentration of 5 mg·mL^−1^ (scale bar = 100 nm), (**b**) TEM image of drug-loaded (5 wt.% Dox·HCl) polymersomes (scale bar = 100 nm) [80].

For a visual illustration of a drug-loaded polymersome, Figure 3 depicts transmission electron microscopy (TEM) images, showcasing the structural integrity of blank (Figure 3a) and drug-loaded polymersomes (Figure 3b) designed for targeted drug delivery of DOX [80].

## 3. Advantages and Limitations of Polymersomes as Compared with Those of Liposomes

**Figure 4:**
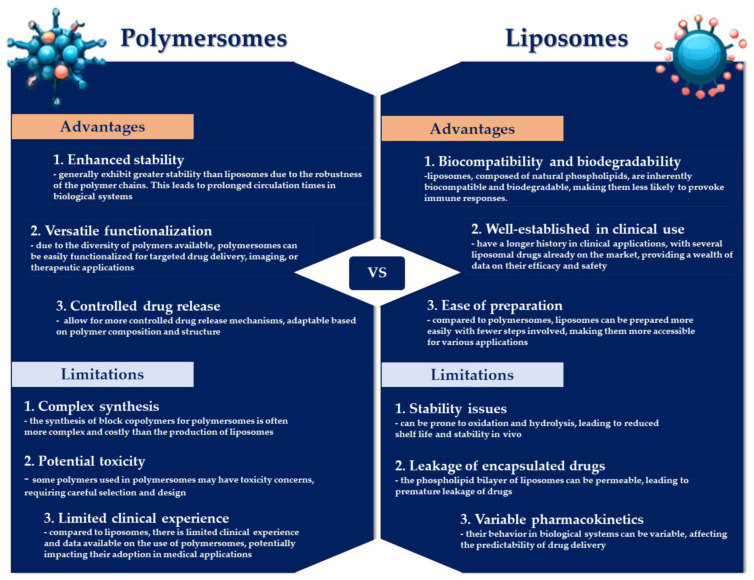
Advantages and limitations of polymersomes as compared with their counterparts, liposomes.

Both polymersomes and liposomes possess distinct benefits and limitations (Figure 4). Liposomes are highly regarded as drug delivery systems, being systematically investigated for the conveyance of various therapeutic agents [44]. A notable limitation of liposomes lies in their inadequate biochemical and mechanical stability when exposed to complex biological fluids, such as blood, potentially leading to the premature and unregulated discharge of the therapeutic contents [81]. In contrast, polymersomes exhibit superior stability relative to their lipid-based counterparts, attributable to some differences in membrane thickness, lateral diffusivity, and molecular entanglement [82]. However, the manifestation of these properties is contingent on several factors, including the size of the polymeric vesicles, their fabrication method, the characteristics of the amphiphiles, and the conditions under which they are stored [81]. The bilayer membrane of polymersomes is considerably thicker (~50 nm) compared to that of liposomes (~3–5 nm), enabling the incorporation of larger quantities of hydrophobic drug molecules within polymersomes’ more substantial hydrophobic bilayer [44,83]. However, polymersomes exhibit a reduced capacity for encapsulating hydrophilic molecules, which curtails their utility as drug carriers, especially in the context of anticancer treatments [84]. Despite the enhanced durability and stability of the polymersomes’ bilayer membrane, these attributes can also present disadvantages [85]. Nevertheless, the chemical adaptability of polymersomes facilitates the programmable release of cargo through various external and internal stimuli. Yet, the relatively impermeable nature of polymersomes towards macromolecules, ions, and small molecules represents a limitation that may impede their biomedical applications [75,86]. Liposomes are more biocompatible than polymersomes, which are synthesized from synthetic components, leading to slower degradation and increased toxicity levels in polymersomes compared to liposomes [87]. Alibolandi M. et al. explored the distribution and therapeutic effectiveness of doxorubicin-loaded polymersomes (Poly-DOX) versus a liposomal formulation in a murine colon adenocarcinoma model, finding that Poly-DOX preferentially accumulated in the tumor and displayed significantly reduced liver concentrations 48 h after treatment compared to the liposomal version. Notably, the dose tolerated by mice treated with Poly-DOX was significantly lower, yet it offered superior therapeutic efficacy in reducing tumor growth rate, suggesting a potential for minimizing adverse side effects [88]. Comparative studies on the pharmacokinetics of DOX-loaded polymersomes (PolyDoxoSomes) and commercially available liposomal DOX (Lipo-DOX) revealed that PolyDoxoSomes had a shorter plasma half-life and a lower area under the curve (AUC), which might contribute to lesser side effects and dose-related toxicities. Despite these differences, the therapeutic outcomes of the polymersomal formulation were on par with those of Lipo-DOX [89].

Zou et al. developed redox-sensitive, DOX-loaded polymersomes decorated with the cNGQGEQc peptide, which demonstrated extended circulation times and significantly enhanced tumor targeting in mice compared to Lipo-DOX, alongside a substantially higher maximum-tolerated dose [90]. Further, Youssef S. F. et al. assessed the in vivo pharmacokinetics of flutamide-loaded polymersomes versus a liposomal formulation following oral administration, revealing that the polymersomal variant resulted in markedly higher plasma concentrations than both the liposomal and drug suspension forms [91]. Additionally, GE11 peptide-modified polymersomes containing DOX, as investigated by Zou Y et al. for treating SKOV3 human ovarian tumors, showed significantly greater tumor accumulation and effectively hindered tumor progression with lower toxicity compared to Lipo-DOX, as evidenced by in vivo biodistribution studies and in vitro toxicity assessments [92].

These studies underline the nuanced pharmacokinetic profiles and biodistribution patterns of polymersomal formulations in comparison to liposomal counterparts, highlighting their potential for enhanced therapeutic efficacy and reduced side effects in cancer treatment.

## 4. Stimuli Responsiveness of Polymersomes

Stimuli-responsive polymersomes are dynamic drug carriers capable of detecting and reacting to a range of stimuli. These stimuli can be classified as: (i) internal biological stimuli, including redox potential, enzymatic reactions, pH, etc., and (ii) external physical stimuli, such as electric fields, light, ultrasound, mechanical force, temperature, etc. [93]. In their design, these nanocarriers are equipped with chemical groups which are sensitive to specific stimuli [93]. Upon exposure to these triggers, the nanocarriers experience chemical or physical changes, such as disassembly, bond cleavage, membrane fusion, and swelling, among others [93]. These stimuli-responsive polymersomes are tuned to detect minimal variations in surrounding factors like pH, light intensity, enzyme and ion concentrations, gases, mechanical forces, temperature, and redox agents [93]. They can “translate” various environmental changes into functional responses, enhancing membrane permeability, altering morphology, and causing changes in volume. This adaptability is achieved through physicochemical modifications of the chain networks and membranes.

### 4.1. pH Responsiveness

The general mechanism of pH-responsive materials involves their ability to undergo structural or compositional changes in response to variations in pH levels. These materials are engineered to be sensitive to the acidic environments often found within tumor tissues or specific cellular compartments (such as endosomes and lysosomes), which typically exhibit lower pH values compared to the normal physiological pH of 7.4 [94]. It is important to mention that the tumor microenvironment has been shown to have pH levels as low as 4.5 [95].

In the context of cancer therapy, pH-responsive materials exploit the acidic microenvironment of tumors, which can have a pH ranging from 6.5 to 7.2 [94], or even lower inside cellular compartments, to trigger therapeutic actions such as drug release. The mechanism works as follows:

(i) Protonation/deprotonation: at different pH values, the ionizable groups within the material can accept or donate protons (H^+^ ions), leading to a change in their charge state. This change can alter the material’s solubility, leading to disassembly or swelling of the carrier, and thus the release of the encapsulated drug [96].
(ii) Hydrolysis: certain chemical bonds within the material may be stable at neutral pH but become susceptible to hydrolysis under acidic conditions. This can lead to the degradation of the material and subsequent drug release [97].
(iii) Conformational change: polymers may undergo a change in their conformation in response to pH, transitioning between expanded and collapsed states. This shift can expose or hide drug molecules, controlling their release based on the environmental pH [98].

By leveraging these mechanisms, pH-responsive materials can ensure that drugs are released specifically at the site of the tumor or within target cells, enhancing the efficacy of the treatment while minimizing systemic side effects. This specificity makes pH-responsive polymersomes and other nanocarriers particularly attractive for the targeted delivery of cancer therapeutics.

pH-responsive polymersomes function by utilizing the pH gradient between the tumor microenvironments and the surrounding unaffected tissues [94]. These types of polymersomes are designed with structures containing acid-cleavable bonds and linkers, such as hydrazine, ortho esters, acetal, and imine [99].

In a recent report, a polyplex polymersome decorated with histatin 5 (Hst5) is proposed as a smart doxorubicin (DOX) nanocarrier for targeted treatment and imaging of cancer cells. The approach consists of the synthesis of a methoxy (polyethylene glycol)-b-(polycaprolactone)-based polymersome (mPEG-b-PCL). It was tested for its pH-dependent drug release and effect on cancer cells and imaging by means of fluorescence. The data demonstrate a pH-induced drug release in the presence of Hst5. This was further shown through cell culture data where Hst5 increased its effect by 26–41% [100].

In another recent study, an amphiphilic triblock copolymer methoxyl poly(ethylene glycol)-block-poly(L-lysine)-block-poly(2-(diisopropyl amino)ethyl methacrylate) (abbreviated as mPEG-PLys-PDPA or PLD) consisting of a hydrophilic diblock mPEG-PLys and a hydrophobic block PDPA is synthesized. The as-obtained polymersome can efficiently codeliver DOX as a hydrophilic chemotherapeutic model and siRNA against ADP-ribosylation factor 6 (siArf6) as an siRNA model into cancer cell via lysosomal pH-triggered payload release. PC-3 prostate cells are synergistically killed by the DOX- and siArf6-coloading polymersome (namely PLD@DOX/siArf6) [101].

The strategy of employing polyacid and acid-cleavable bonds in drug delivery systems has seen a decline. Recent trends in the literature show a growing preference for polybase-bearing blocks such as poly(2-(diisopropylamino)ethyl methacrylate) (PDPA), poly(2-(diethylamino)ethyl methacrylate) (PDEAEMA), 2-(pentamethylene imino)ethyl methacrylate (PEMA), and poly-β-amino esters. This shift is largely attributed to their relatively low pKa values, which are well-suited to the pH of healthy tissues. In the bloodstream, where the pH is ~7.4, the tertiary amine groups present in the polymersome membrane remain neutral, rendering the polymersomes impermeable [26]. However, upon endocytosis by cells or when exposed to the acidic tumor microenvironment, these tertiary amine groups become protonated, transforming into positively charged quaternary ammonium groups. This change leads to a significant increase in the hydrophilicity of the hydrophobic block, resulting in the disassembly or poration of the membrane [26]. This pH-responsive behavior is particularly advantageous for targeted drug delivery, as it allows for the controlled release of therapeutic agents in response to the specific acidic conditions of tumor sites, thereby enhancing the efficacy of the treatment while minimizing side effects on healthy tissues [26]. Additional structures used in pH-responsive polymersomes include hydrolytic polymers [17], polymers with ionizable groups [102], and oppositely charged block copolymers [103]. These bonds and linkers are highly sensitive to acidic conditions, making them prone to rapid hydrolysis, leading to swelling, disassembly, and the targeted release of drugs at tumor sites.

### 4.2. Temperature Responsiveness

Temperature-sensitive polymersomes are made from polymers that change their structure or undergo a phase transition when exposed to different temperatures. These transitions affect the polymer’s hydrophilic–hydrophobic properties, as well as its solubility, leading to the creation or breakdown of these structures [104]. Certain polymers used in drug delivery can become insoluble when heated past a low critical solution temperature (LCST), while others have a high critical solution temperature (UCST) and dissolve upon heating [105]. This feature is particularly useful in cancer treatments since tumor areas tend to be warmer than surrounding healthy tissue. Additionally, cooling the area with external methods like ice packs or cryoprobes can trigger these polymersomes to release their drug cargo [106,107]. Poly(N-isopropyl acrylamide) (PNIPAM) serves as a quintessential model of a thermoresponsive polymer, showcasing pronounced volumetric transitions across the lower LCST threshold of 32 °C [108]. Below this LCST, PNIPAM exhibits a highly swollen configuration. Conversely, exceeding this temperature threshold prompts a shift to a dehydrated and collapsed state, facilitating the release of encapsulated drugs [109]. Although PNIPAM’s inherent LCST falls beneath human body temperature, the incorporation of hydrophilic copolymer monomers has been demonstrated to elevate this threshold, rendering it more compatible with physiological conditions.

A study introduces an innovative triblock copolymer polymersome, designed for the controlled release of DOX at body temperature, specifically within the range of 37–42 °C. Employing controlled RAFT polymerization, the temperature-sensitive poly(N-vinylcaprolactam)n-poly(dimethylsiloxane)_65_-poly(N-vinylcaprolactam)_n_ copolymers are synthesized with varying chain lengths, achieving monodispersity and stable vesicle formation at ambient temperatures. The permeability of these polymersomes, encapsulating DOX, was meticulously modulated by the copolymer’s chain length in response to slight temperature variations, facilitating sustained drug delivery. This capability, coupled with their high drug-loading efficiency and reversible, temperature-induced morphological changes without compromising structural integrity, positions these polymersomes as promising candidates for stimuli-responsive drug delivery systems in cancer treatment [110].

### 4.3. Reduction Responsiveness

A particular category of stimuli-responsive polymers is engineered to react to reducing agents, such as glutathione (GSH), which are prevalent within the cytosolic environments of neoplastic cells [111].

Polymers that are biochemically reducible incorporate disulfide linkages within their molecular architecture and have been extensively employed for applications in gene silencing, gene delivery, and siRNA delivery [112]. These bioreducible stimuli-responsive polymers possess a disulfide bond (–S–S–) either within the core or along the side chains of the amphiphilic polymer, demonstrating stability under the oxidizing conditions found outside the cell despite the bond’s relatively lower dissociation energy compared to carbon–carbon (–C–C–) bonds [112]. The thiol-disulfide exchange reactions with GSH facilitate the reduction of disulfide bonds in intracellular settings. Polymers featuring disulfide bridges are thus readily degradable, enabling a more efficient release of therapeutic agents within the cellular milieu as opposed to their stability in the circulatory system [112]. This degradation is largely attributed to the elevated concentrations of GSH in intracellular compartments (such as the cytosol, mitochondria, and cell nucleus) in comparison to extracellular fluids [99]. The intracellular environment, characterized by a significant reducing capacity due to the predominant localization of GSH (85–90%) in the cytosol relative to other organelles (which contain only 10–15% of GSH) [113], establishes a concentration gradient that is critical for the development of reduction-responsive polymersomes with disulfide bonds, thereby enabling targeted drug delivery to specific sites of action [113].

**Figure 5:**
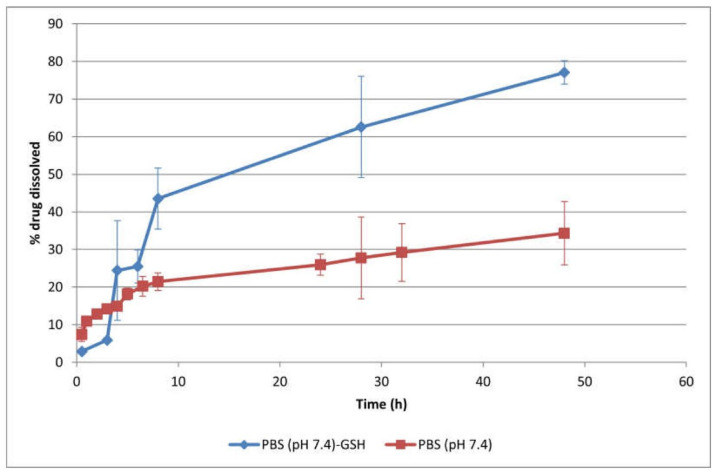
Cumulative in vitro release profiles of drug-loaded (10 wt.% Dox·HCl) polymersomes in PBS (10 mM, pH 7.4) with or without 50 mM GSH at 37 °C (mean ± SD, *n* = 3). The polymersomes exhibited a prolonged drug release behavior, with 34.3 ± 8.4% drug released at physiological pH after 48 h, attributed to their structural stability. The drug release rate increased at 50 mM GSH, with a cumulative drug release up to 77.1 ± 3.1% after 48 h. The disulfide linkages in the hydrophobic block of the copolymer were cleaved in the reductive environment, leading to the rupture of polymersomes, which subsequently accelerated drug release [80].

Polymersomes were effectively synthesized through the self-assembly of the amphiphilic, biodegradable mPEG–PDH–mPEG triblock copolymer in phosphate buffer solution (pH 7.4, 10 mM) at ambient temperature. Utilizing an optimized self-assembly technique, the anticancer drug Dox·HCl was encapsulated into the hydrophilic core of the polymersomes (10 wt.% feeding ratio), achieving an impressive encapsulation efficiency of 98% wt.%. The polymersomes, measuring 124 nm, were suitably sized for passive targeting through the EPR effect, while their negative zeta potential (−24 mV) indicated their colloidal stability. These polymersomes exhibited sustained drug release in physiological settings, predominantly regulated by the drug’s diffusion across the polymeric boundary. Additionally, the polymersomes’ redox-responsive attribute expedited DOX (Figure 5) release under reductive conditions (50 mM GSH) due to a synergistic effect of diffusion and erosion [80].

Yang et al. developed reduction-responsive chimeric polymersomes (RCPs) that were functionalized with the CC9 peptide, aimed at the efficient delivery of pemetrexed disodium (PEM) to H460 human lung tumor cells both in vivo and in vitro [114]. The CC9-functionalized RCPs encapsulating PEM demonstrated a hydrodynamic size of approximately 60 nm, alongside a notable increase in PEM-loading capacity (by 14.2 wt.%) and an optimal CC9 peptide density of 9.0%. The release of PEM from these polymersomes was significantly facilitated by the presence of GSH within tumor cells, indicating a high responsiveness to the reductive tumor environment. The PEM-loaded RCPs targeted with CC9 exhibited markedly superior antitumor efficacy against H460 tumor cells, as compared to PEM-RCPs lacking CC9 and free PEM, respectively. Furthermore, when compared to clinical formulations of PEM, the PEM-CC9-RCPs achieved a considerably longer circulation time (increased by 22-fold) and a higher accumulation rate of PEM within H460 cancer cells (enhanced by 9.1-fold). The incorporation of the CC9 peptide into the polymersomes significantly improved their penetration into tumor cells, leading to an effective inhibition of H460 xenograft growth and an extension of survival times in the treated mice [114].

Recently, a biodegradable, redox-responsive triblock copolymer, denoted as mPEG–PDH–mPEG, was engineered, featuring a central hydrophobic segment embedded with disulfide bonds flanked by two hydrophilic poly(ethylene glycol) methyl ether sections. Notably, Dox·HCl was successfully encapsulated into these polymersomes with an exceptional efficiency rate (up to 98 wt.%). In vitro assessments of drug release revealed a sustained and diffusion-controlled release under physiological conditions, with approximately 34% of the drug released after 48 h. The presence of 50 mM GSH prompted the cleavage of disulfide bonds, significantly enhancing drug release (up to ~77%) through an erosion-driven mechanism. Consequently, these meticulously designed polymersomes emerge as promising vehicles for the targeted delivery of therapeutic agents within the reductive situation of cancerous cells [80].

Recent investigations have elucidated the development of polymersomes sensitive to oxidative conditions, facilitating targeted delivery of therapeutic agents. The presence of reactive oxygen species (ROS), H_2_O_2_, or oxidative stress at tumor sites prompts the disintegration of these polymeric structures. Li et al. have synthesized polymersomes loaded with glucose oxidase, employing diblock copolymers composed of PEG and a copolymer derived from camptothecin (CPT) and piperidine-modified methacrylate [115]. The acidic milieu prevalent within tumor areas instigates H_2_O_2_ production. Elevated H_2_O_2_ levels catalyze the self-decomposition of the polymersomes, consequently liberating CPT. This release mechanism inhibits tumor proliferation via a synergistic effect, underscoring the potential of such oxidation-sensitive polymersomes in cancer therapy [115].

### 4.4. Enzyme Responsiveness

Enzyme-responsive polymersomes have emerged as a cutting-edge category of polymeric vesicles. The core motivation behind their development is that the enzyme levels are often elevated in a range of pathological states, including cancer, inflammation, and thrombosis [116]. These elevated enzyme levels serve not only as biomarkers for diagnosis but also play a crucial role in therapeutic interventions [116]. The construction of these polymersomes involves the strategic incorporation of enzyme-sensitive moieties into the copolymer structure through stable covalent linkages [116]. When these polymersomes encounter specific enzymes prevalent in diseased tissues [116], they undergo distinct physicochemical transformations, enabling them to dynamically respond to the pathological environment. This responsiveness to enzymatic activity allows for the selective release of encapsulated drugs at the site of disease, leveraging the abnormal enzyme concentrations as triggers for therapeutic intervention.

In an illustrative study, Ramazani et al. developed enzyme-responsive polymersomes for the delivery of the hydrophobic drug SN38, targeting nucleolin-positive C26 colorectal cancer cells. The innovative approach involved conjugating PEG to PLA through the intermediary peptide PVGLIG, a sequence recognized for its specificity to be cleaved by matrix metalloproteinase-2 (MMP-2), a tumor-associated enzyme. The resulting polymersome, characterized by its spherical form with an average diameter of 172 ± 30 nm, demonstrated an impressive SN38 encapsulation efficiency of approximately 70.3% ± 3.0%. Under physiological conditions, the release rate of SN38 was meticulously controlled; however, upon exposure to MMP-2, a significant enhancement in drug release was observed, increasing by approximately sevenfold, which underscored the enzyme-responsive capability of the polymersome platform [117].

Enzyme-responsive polymersomes are engineered to provide controlled drug release, leveraging the stability of their bilayer structures to maintain sustained release profiles in enzyme-deficient environments. This stability is attributed to the rigid bilayer composition of polymersomes, which acts as a barrier to early drug release. However, the introduction of hydrolytic enzymes in vivo initiates a targeted degradation process of the polymersomes’ cleavable components, such as peptides, that have been strategically incorporated into the nanocarrier’s design. This interaction with enzymes triggers either functional or structural modifications within the polymersomes, enabling a responsive release mechanism [118,119].

The cleavage of bonds within the Ps, induced by enzymatic activity, predominantly occurs in three critical regions:

(i) At the hydrophobic membrane: this layer, responsible for the structural integrity and encapsulation capacity of the polymersomes, is particularly vulnerable to enzymatic cleavage, leading to the disintegration of the membrane and subsequent release of the encapsulated agents [118,119].
(ii) At the hydrophilic brush: the hydrophilic brush, which extends into the aqueous environment and contributes to the polymersome’s solubility and stability, can also undergo enzymatic degradation, affecting the polymersome’s interaction with the surroundings and its release kinetics [118,119].
(iii) At the link between the hydrophilic brush and hydrophobic membrane: the junctions between these two structural components are crucial for the polymersome’s architecture. Enzymatic cleavage here can lead to a significant alteration in the polymersome’s configuration, directly impacting drug release rates and profiles [118,119].

Yao et al. have contributed to the field of enzyme-responsive drug delivery by elucidating the role of the endogenous enzyme NAD(P)H:quinone oxidoreductase isozyme 1 (NQO1) in activating polymersomes designed for photodynamic therapy (PDT). These polymersomes were uniquely constructed with quinone-bridged photosensitizers, specifically Nile Blue and coumarin, integrated into the hydrophobic membrane and further stabilized by quinone trimethyl lock-capped self-immolative side linkages. In the absence of NQO1, a “double-quenching” mechanism ensures that both the photodynamic therapeutic effect and fluorescence emissions remain in an inactive, or “off”, state, effectively preventing premature activation or toxicity. Upon cellular uptake by cancer cells, where NQO1 is often overexpressed, the enzyme catalyzes the self-immolative cleavage of the quinone trimethyl locks. This enzymatic action triggers a controlled release of the encapsulated photosensitizers—coumarin and Nile Blue. The release mechanism transitions the near-infrared (NIR) emission from an “off” to an “on” state, thereby activating the photosensitizers for photodynamic therapy. This innovative approach leverages the specific overexpression of NQO1 in cancer cells to achieve targeted activation of PDT, minimizing damage to surrounding healthy tissues and enhancing the therapeutic efficacy of the treatment [120].

### 4.5. Hypoxia Responsiveness

Hypoxia, a prevalent characteristic observed in ~60% of solid tumors, stands as a significant hallmark within the microenvironments of solid tumor tissues [121,122]. Typically, the partial pressure of oxygen (pO_2_) in healthy tissues ranges from 40 to 60 mmHg (50–80 μmol/L), in stark contrast to tumor tissues where pO_2_ falls below 10 mmHg (13 μmol/L), with certain observations reporting levels as diminished as 0–2.5 mmHg [123,124,125]. This pathological state arises from the disproportionate oxygen consumption by tumor cells relative to the supply delivered through the bloodstream. The underlying causes include (1) the intensified metabolism and cellular proliferation in cancer cells, leading to heightened oxygen consumption, (2) the chaotic tumor vasculature contributing to an inadequate oxygen supply, and (3) the limited diffusion capacity of oxygen (<200 μm) which fails to satisfy the oxygen demands of cancer cells situated distally from blood vessels [126,127].

Tumors characterized by hypoxia typically demonstrate increased aggressiveness, reduced responsiveness to therapeutic interventions, and a poorer prognosis in treatments such as chemotherapy, radiotherapy, and photodynamic and sonodynamic therapy [128,129]. The phenomenon of intratumoral hypoxia leads to the hyperactivation of hypoxia-inducible factor-1 (HIF-1), a key regulator in cancer metastasis and resistance to radiotherapy and chemotherapy [128]. Furthermore, cancer cells adapted to hypoxic conditions often exhibit slower rates of division, rendering them less susceptible to chemotherapeutic agents that target DNA replication processes [128]. Additionally, oxygen is essential for repairing DNA damage induced by radiation therapies (e.g., X-rays, γ-rays, electron beams) and acts as a precursor for the generation of reactive oxygen species (ROS) that are instrumental in eradicating cancer cells in photodynamic and sonodynamic therapies. Consequently, the hypoxic microenvironment within tumors significantly impedes the efficacy of radiotherapy, photodynamic, and sonodynamic therapy, leading to treatment resistance and diminished therapeutic outcomes [130,131,132]. While hypoxia is often viewed as a negative prognostic factor in cancer treatment, the distinct biological attributes of the microenvironment in hypoxic cancer tissues provide a specialized avenue for targeted antitumor therapies. This is partly due to the Warburg effect, whereby cancer cells exhibit a preference for aerobic glycolysis over the more efficient oxidative phosphorylation for their metabolic needs. Consequently, this metabolic shift leads to the upregulation of several enzymes involved in reduction reactions or electron donation in hypoxic conditions, such as nitroreductase, azoreductase, inducible nitric oxide synthase, methionine synthase reductase, DT-diaphorase (DTD), and nicotinamide adenine dinucleotide phosphate (NADPH) [133,134]. Leveraging these insights, scientists have engineered hypoxia-activated prodrugs that are selectively activated by these enzymes, offering a strategic approach to treating hypoxic tumors [135].

Despite the conceptual promise of hypoxia-activated prodrugs, including their defined molecular structures and predictable pharmacokinetics, their therapeutic efficacy has often been below expectations. This shortfall is attributed to the rapid clearance of these small-molecule drugs from the bloodstream through renal filtration, which leads to suboptimal drug concentrations at the tumor site. Furthermore, the necessity for high doses to achieve therapeutic levels exacerbates the risk of systemic toxicity and adverse side effects [133].

In a study by Mamnoon et al., polymersomes conjugated with 17β-estradiol were designed to respond to hypoxic conditions for diminishing the viability of estrogen-receptor-positive breast cancer cells. These targeted polymersomes (E_2_-Dox-HRPs) were observed to notably decrease the size of MCF7 spheroids under hypoxic environments, outperforming the results seen with non-targeted polymersomes and unencapsulated drugs. Thanks to the incorporation of 17β-estradiol on their exterior, these polymersomes have the capacity to attach to estrogen receptors on breast cancer cells, facilitating their internalization. They demonstrated an efficiency of 59% in encapsulating DOX. The integration of a hypoxia-responsive element within the polymer structure permits the targeted release of DOX directly into hypoxic breast cancer cells, thereby amplifying the drug’s therapeutic impact against ER-positive cancer cells. The distinct advantages of the targeted polymeric nanoparticles include their specificity for estrogen receptor-positive breast cancer cells, penetration into the hypoxic core of microtumors, controlled drug discharge, and the consequent reduction in cancer cell viability. Pending further advancements, these 17β-estradiol-conjugated, hypoxia-responsive polymersomes exhibit significant promise for precision drug delivery in the treatment of estrogen-receptor-positive breast cancer [136].

In another study, polymersomes functionalized with iRGD peptide and responsive to hypoxic conditions effectively encapsulated the anticancer drug gemcitabine with a 50% efficiency rate. These polymersomes demonstrated the capacity to release their contents both in vitro and in vivo, significantly enhancing penetration depth while reducing cell viability under hypoxic conditions. Notably, these polymersomes exhibited echogenic properties, facilitating their use in ultrasound imaging. In vivo imaging studies verified their enhanced targeting and release capabilities within the hypoxic tissues of mice bearing xenograft tumors derived from pancreatic cancer cells. Our findings highlight the potential of echogenic, iRGD-decorated, hypoxia-responsive polymersomes as dual-functional tools for imaging hypoxic regions and delivering therapeutic agents directly to the hypoxic areas of pancreatic tumors [137].

### 4.6. Light Responsiveness

Electromagnetic irradiation serves as a remote and spatio-temporal modulator for the controlled dispensation of therapeutic agents from nanostructures, either through direct or indirect mechanisms to facilitate phototherapeutic interventions. In spite of the electromagnetic spectrum encompassing irradiation wavelengths from the longest, such as radio and microwave (MW), to the shortest, including X-rays and gamma-rays, only ultraviolet (UV) irradiation (10 nm < λ < 400 nm) and near-infrared (NIR) irradiation (760 nm < λ < 1500 nm) are presently employed as stimuli for the liberation of payloads from drug delivery systems [138].

The utilization of light as a trigger offers several advantages, including the ability to precisely control the timing and location of drug release with minimal invasiveness [139]. This specificity is particularly valuable in reducing systemic side effects and enhancing the efficacy of the encapsulated drugs [140]. Moreover, the adjustable nature of the photoresponsive elements allows for customization of the polymersomes to release their cargo under specific light conditions, enabling applications across a wide range of therapeutic areas.

Photoresponsive polymersomes are engineered to utilize light as an external stimulus for drug delivery, incorporating photosensitive moieties within their amphiphilic block polymers to achieve controlled release of therapeutic cargo at targeted sites. Polymersomes are primarily constructed through the self-assembly of amphiphilic copolymers, which incorporate light-activatable entities such as functional chromophores, photosensitizers, photothermal conversion agents, and light-responsive nanoparticles [141]. These light-activatable components may be covalently bonded to the amphiphilic copolymer or enclosed within any compartment of the polymersome. Upon exposure to light, these moieties absorb the radiation and subsequently transform it into a chemical signal through various mechanisms, including photoreactions, photosensitization-induced oxidation, thermal generation, and photoconversion. These processes lead to the disintegration and rupture of the polymersome structure, thereby facilitating the targeted release of encapsulated agents. The therapeutic agent release mechanism of the light-responsive polymersomes is induced by light-mediated photoreactions of chromophores, the photothermal effect, photo-oxidation, upconversion processes, and multi-functional controlled release [27]. These mechanisms are reviewed elsewhere [27].

**Figure 6:**
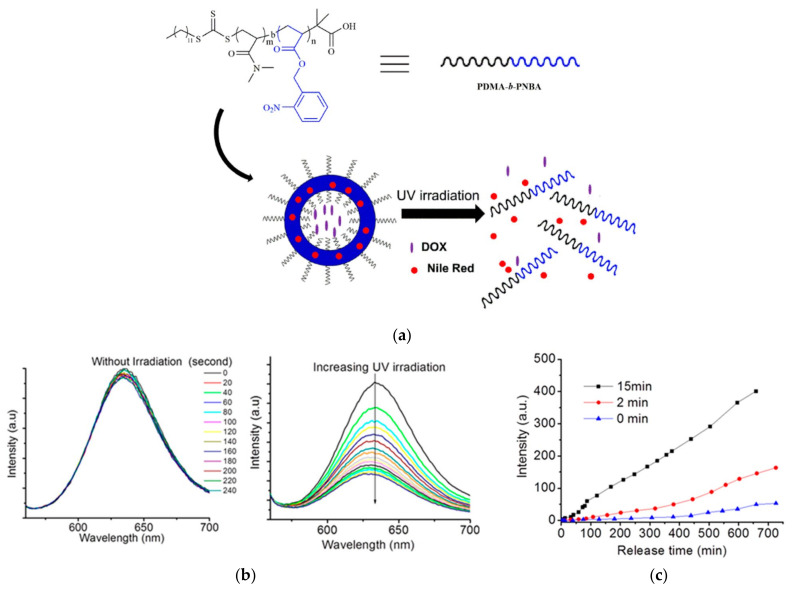
(**a**) Schematics for the controllable light-responsive corelease of DOX and NR drugs from the developed polymersomes of PDMA-b-PNBA. PDMA-b-PNBA: N,N′-dimethylacrylamide-b-o-nitrobenzyl acrylate; (**b**) (**left**)—emission spectra of NR for the polymersome solution without UV irradiation; (**right**)—emission spectra of NR for the polymersome solution under UV irradiation at 365 nm; (**c**) controlled release of DOX on exposure to UV light with different times [35].

Hou and colleagues developed a methodology for synthesizing amphiphilic block copolymers comprising poly(N,N′-dimethylacrylamide) (PDMA) as the hydrophilic segment and poly(n-butyl acrylate) (PNBA) as the hydrophobic component, designated as PDMA-b-PNBA. This synthesis employed bulk reversible addition–fragmentation chain transfer (RAFT) polymerization utilizing 2-(Dodecylthiocarbonothioylthio)-2-methylpropionic acid as the macro-RAFT agent. To assess the capability for phototriggered drug release, the polymersomes, self-assembled via the emulsion technique, were coloaded with DOX and Nile Red (NR) dye. Upon exposure to UV light at 365 nm for 15 min, these polymersomes exhibited a pronounced photoresponsive behavior (Figure 6). The photocleavage reaction of the o-nitrobenzyl (ONB) groups within the hydrophobic block converted it into a hydrophilic entity, leading to the polymersomes’ disassembly and the concurrent release of both DOX and NR. The extent of DOX release from the polymersomes was directly influenced by the duration of UV irradiation [35].

Soo Kim et al. developed a novel approach for intracellular gene knockdown by creating a photoreactive oligodeoxynucleotide (PRO)-embedded vesicular polyion complex (PIC), termed PROsome. This innovative structure is designed to carry the nuclear-enriched abundant transcript 2 (NEAT2)-targeting antisense oligonucleotide (asNEAT2) as a therapeutic agent. The PROsomes’ ability to toggle between crosslinked and decrosslinked states upon exposure to specific UV wavelengths demonstrates their photoswitchable capacity for controlled ASO release. Furthermore, the efficacy of these structures as delivery vehicles was assessed in a biological model using human lung cancer cell culture (A549 cells). The gene knockdown efficiency of X-PROsomes targeting NEAT2—a gene highly expressed in various human cancers, including lung cancer—was evaluated following 0.5 min of UV312 irradiation. The results indicated an impressive gene knockdown efficiency of up to 80% at 48 h postincubation, showcasing the potential of PROsomes for phototriggered enhanced gene knockdown in therapeutic applications [142].

In a recent study, a polymersome is prepared by self-assembling amphiphilic diblock copolymer P(OEGMA-co-EoS)-b-PNBOC and encapsulates the hypoxia-activated prodrug AQ4N and upconversion nanoparticle (PEG-UCNP) in its hydrophilic cavity [143]. Thirty minutes of NIR preactivation triggers crosslinking of NBOC and converts the permeability of the polymersome with sustained AQ4N release until 24 h after the NIR preactivation. The photosensitizer EoS is activated and exacerbates environmental hypoxic conditions during a sustained drug release period to boost the AQ4N therapeutic effect. The combination of sustained drug release with concurrent hypoxia intensification results in a highly efficient tumor therapeutic effect both intracellularly and in vivo. This biomimetic polymersome will provide an effective and universal tumor therapeutic approach.

However, there is a valid concern regarding the potential for DNA damage when applying UV light, especially in the context of cell culture studies. UV radiation, particularly at shorter wavelengths (UVC and UVB), has been well-documented to cause various types of DNA damage, such as the formation of cyclobutane pyrimidine dimers and pyrimidine (6-4) pyrimidone photoproducts [144], which can lead to mutations and cellular toxicity if not adequately repaired. This presents a significant limitation in the use of UV light for in vitro applications, where direct exposure to cells could potentially compromise cell viability and affect experimental outcomes. Moreover, the penetration depth of UV light in biological tissues is relatively shallow, limiting its utility for in vivo applications where deeper tissue penetration is required for therapeutic efficacy [145]. This limitation is particularly relevant for the treatment of conditions located beyond the surface of the skin or for internal tumors.

## 5. Multi-Responsive Polymersomes for Cancer Therapy

Depending on the envisaged application, several polymersome-based nanocarrier systems are designed to be responsive to multiple stimuli, enabling them to react to various triggers simultaneously.

Dual-responsive polymersomes have emerged as a groundbreaking innovation in cancer therapy, offering targeted and controlled drug delivery by responding to specific physiological stimuli. These nanoscopic vesicles exhibit sensitivity to variations of the surrounding tumoral media and external stimuli, making them ideal for the precise delivery of chemotherapeutics directly to tumor sites. Upon reaching the targeted area, these polymersomes can release their encapsulated drug cargo in response to the slightly acidic environment of tumor tissues or the elevated temperatures associated with hyperthermia treatment. This unique dual-responsive capability ensures that drugs are released more efficiently and selectively within the tumor microenvironment, minimizing the impact on healthy cells and reducing systemic side effects. Below, we have collected various works (Table 3) on the complex nature of dual-responsive polymersomes. Each entry highlights a specific combination of polymers forming the polymersomes, their responses to stimuli, the active pharmaceutical ingredient they are designed to deliver, the cancer type they target, and the outcome of their application. This framework provides a concise overview of how dual-responsive polymersomes can be tailored to enhance cancer therapy’s specificity and efficacy.

There are polymersomes that are both photocrosslinked and sensitive to temperature and pH, used for the codelivery of DOX and PTX [146]. Others include ultraviolet and redox responsiveness for the delivery and release of DOX [148], and redox- and pH-responsive polymersomes containing ferrocene moieties [149]. These multi-stimuli-responsive polymersomes are particularly wanted for treating complex cancers like glioblastoma due to factors like multi-drug resistance, hypoxia in the tumor microenvironment, and tumor heterogeneity.

Kozlovskaya et al. synthesized dual-responsive triblock copolymers, namely poly(N-vinylcaprolactam)_10_-b-poly(dimethylsiloxane)_65_-b-poly(N-vinylcaprolactam)_10_ (PVCL_10_-PDMS_65_-PVCL_10_) and PVCL_5_-PDMS_30_-PVCL_5_, and subsequently assembled them into polymersomes in aqueous solutions via the nanoprecipitation method. The temperature-responsive vesicles, specifically PVCL_10_-PDMS_65_-PVCL_10_ and PVCL_5_-PDMS_30_-PVCL_5_, were found to encapsulate the anticancer drug DOX with high loading efficiencies of 40% and 34%, forming spherical polymersomes with average diameters of 470 and 360 nm, respectively. DOX was predominantly released at an acidity level between pH 4 and 3, with a 97% cumulative release achieved within 24 h at pH 3. The in vivo toxicity and biocompatibility of PDMS65-PVCL_10_ vesicles were also assessed through intravenous injection (40 mg kg^−1^ single dose) into C57BL/6j male mice. The subacute toxicity study, spanning 14 days, included gravimetric, histological, and hematological analyses, which showed no significant weight loss, stable organ weight/body weight ratios, blood cell counts within normal ranges, and no apparent pathological changes in major organs such as the heart, kidney, and spleen [147].

Zhu et al. reported pH- and hyperthermia-responsive polymersomes containing ICG, DOX, and NH_4_HCO_3_ for combined chemophotothermal treatment of 4T1-Luc tumor cells in BALB/c mice. The encapsulation efficiency (EE) and loading efficiency (LE) of these polymersomes (BG-DIPs) for DOX were 14.55 ± 1.61% and 3.49 ± 0.21%, respectively, and for ICG they were 82.32 ± 1.37% and 4.23 ± 0.18%. Upon tumor cell internalization, BG-DIPs generate CO_2_ bubbles through NH_4_HCO_3_ decomposition induced by ICG-driven hyperthermia and/or acidic tumor microenvironment, leading to the structural destruction of BG-DIPs and rapid drug release. Interestingly, the IC50 values of BG-DIPs were 11.78 μg/mL and 5.53 μg/mL at 24 and 48 h postincubation, respectively, higher than that of the free drug, indicating the enhanced cytotoxicity of the free DOX. This is attributed to the passive diffusion of free DOX into cells, whereas BG-DIPs are initially internalized through endocytosis before releasing loaded DOX to suppress the tumor [150].

In another work, researchers designed a novel polymersomal prodrug nanoplatform for cancer immunotherapy, which modulates the tumor microenvironment and blocks immune checkpoints to enhance immunogenic cell death. It highlights the encapsulation and efficient release of talabostat (rapidly released at pH 6.8, with or without H_2_O_2_, the cumulative release was as high as 80.9% within 24 h), mesylate, and DOX (cumulative release as high as 93.1% in a pH 5.0 10 mM GSH solution within 24 h) within the tumor, demonstrating significant tumor suppression and immune response activation in vitro and in vivo. Specifically, the study showcases a 60% complete tumor regression ratio in mice, indicating the potential of this “all-in-one” nanoplatform for effective tumor eradication and long-term immune memory against cancer [151].

A notable example by Li et al. involves pH/temperature/reduction-responsive oxygen- and bubble-generating polymersomes (FIMPs) that coencapsulate manganese dioxide (MnO_2_) for oxygen generation, ICG as a hydrophobic photosensitizer, and ammonium bicarbonate (NH_4_HCO_3_) as a bubble-generating agent. The CO_2_ bubbles produced from NH_4_HCO_3_ decomposition under laser irradiation, low pH in the tumor microenvironment, and the cleavage of disulfide bonds in a reducing tumor microenvironment led to the disintegration of the polymersome structure and prompted the release of the cargo. Moreover, the reaction of MnO_2_ with endogenous H_2_O_2_ results in oxygen production (counteracting tumor hypoxia) and ROS production (effectively killing tumor cells) [152].

## 6. Prospects and Challenges of Polymersomes for Clinical Development and Personalized Medicine

Ensuring that drugs encapsulated within nanoparticles (NPs), such as polymersomes, are directly taken up by cells, rather than being prematurely released outside the cells and subsequently internalized, poses a significant challenge. Addressing this challenge necessitates a multifaceted approach, focusing on the design, composition, and functionalization of the NPs, as well as a deeper understanding of the cellular uptake mechanisms.

Functionalizing the surface of polymersomes with targeting ligands that have a high affinity for specific cell surface receptors can enhance the direct uptake of these NPs by target cells. This specificity ensures that the polymersomes are more likely to be internalized by the desired cells, through receptor-mediated endocytosis, before the release of their drug payload [27]. Moreover, incorporating stimuli-responsive elements into the polymersome design can allow for the controlled release of the encapsulated drug in response to specific intracellular cues (e.g., pH changes, enzyme presence). This strategy can minimize premature drug release and ensure that the drug is released only after the polymersomes have been internalized by the target cells [153].

A deeper investigation into the cellular processes governing NP uptake is essential. Different cells may internalize NPs/polymersomes via various pathways, such as clathrin-mediated endocytosis, caveolin-mediated endocytosis, or macropinocytosis. Tailoring polymersomes to exploit these specific pathways can improve direct cellular uptake and minimize extracellular drug release [154]. The size, shape, and surface charge of polymersomes can significantly influence their interaction with cellular membranes and uptake efficiency. By optimizing these properties, researchers can enhance the likelihood that polymersomes are internalized directly by cells, rather than releasing their cargo externally [27,153,154].

Designing polymersomes with biodegradable components that degrade at a controlled rate within the cellular environment can help ensure that the drug is released inside the cells. The degradation rate must be synchronized with the cellular uptake process to minimize external drug release. The commercialization of polymersomes for drug delivery applications faces several significant challenges. One of the primary challenges is the development of scalable, cost-effective manufacturing processes that can produce polymersomes with consistent quality, size, and functional properties. The complexity of polymersome formulations and the need for precise control over their assembly and functionalization can make large-scale production challenging.

Moreover, ensuring the long-term stability of polymersomes during storage is crucial for their commercial success. Factors such as aggregation, degradation, or premature release of encapsulated drugs can affect the efficacy and safety of the final product.

Another challenge is given by the optimization of the drug-loading capacity and achieving controlled, targeted release of therapeutic agents. Despite the advancements in targeting capabilities, achieving precise and efficient targeting remains a challenge. The variability in tumor vasculature, density, and the presence of the tumor microenvironment barriers can hinder the uniform distribution and penetration of polymersomes into the tumor tissue, potentially reducing their therapeutic efficiency. Ensuring that the drug is released in a way that maximizes therapeutic efficiency while minimizing side effects is critical for the success of polymersome-based treatments.

Assessing and ensuring the biocompatibility and reduced immunogenicity of polymersomes is essential. Any adverse immune response can limit their applicability and acceptance as drug delivery vehicles. Polymersomes are typically made from synthetic block copolymers. While many of these materials are designed to be biocompatible and biodegradable, there is still a risk of toxicity. The degradation products of polymersomes must be non-toxic and readily cleared by the body to avoid adverse effects. Moreover, the inherent properties of the polymers used might provoke unintended immune responses, necessitating thorough biocompatibility testing.

Polymersomes can be designed to evade the immune system to reach their target site effectively. However, this evasion must be balanced with their potential use in cancer immunotherapy, where stimulating an immune response is desirable. The design of polymersomes for immunotherapy applications must ensure that they can either deliver immunostimulatory agents effectively or themselves act as adjuvants to provoke a targeted immune attack against cancer cells without inducing systemic inflammation or autoimmunity.

Demonstrating that polymersomes offer a clear advantage over existing drug delivery technologies in terms of cost-effectiveness and patient outcomes is necessary for market acceptance. The development and production costs must be balanced against the clinical benefits they provide.

Navigating the regulatory norms for novel drug delivery systems can be complex and time-consuming. Demonstrating the safety, efficacy, and quality of polymersome-based therapeutics to regulatory authorities requires extensive preclinical and clinical testing, which can be costly and involve significant uncertainty.

The scarcity of polymersome systems in clinical trials raises questions about their slower progress compared to liposomes. A key factor is the nature of their encapsulating material. Liposomes typically utilize widely available natural phospholipids like phosphatidylcholine [155], while polymersomes are predominantly created from synthetic amphiphilic block copolymers [15]. This difference significantly impacts the clinical approval process for polymersomes. Issues such as their toxicity, cellular uptake and release, and the breakdown of polymersomes within the body must be thoroughly understood before they can be approved. Additionally, the wide-ranging molecular weight distribution within the polymeric chains presents further challenges in obtaining regulatory approval.

We found no reports of clinical trials specifically examining the use of polymersome-based nanoplatforms in cancer therapy. Despite this, the exceptional attributes of polymersome systems inspire the development of advanced carriers for drug delivery and cancer diagnosis, pending further clinical exploration of their pharmacokinetic and pharmacodynamic properties. Recent studies have been based on the effectiveness of polymersomes in vivo, paving the way for their potential inclusion in clinical trials. One study highlights the use of tamoxifen (Tam)-loaded polymersomes, modified with the tumor-penetrating peptide iRGD, which targets fibronectin (FN)/β1 integrin interactions in estrogen receptor (ER)-positive breast cancer cell lines (MCF7, T47D, and HC11) [156]. This discovery is significant considering that ~75% of breast cancers are ER+ and over 30% of women treated with Tam experience relapse within 15 years of initial diagnosis. Another research points to the use of iRGD-peptide-decorated, reduction-sensitive polymersomes carrying napabucasin (BBI608, a cancer stemness inhibitor), which effectively target neuropilin-1 receptors overexpressed in human prostate cancer cells and prostate cancer stem cells (PCSCs). These peptides enable the Ps to deeply penetrate microtumors, at least 200 μm [157]. This is crucial because PCSCs, known for their stem cell-like properties such as self-renewal and multi-lineage differentiation, contribute significantly to tumor heterogeneity, leading to metastasis, recurrence, and drug resistance. The tumor microenvironment, characterized by cellular mutations and epigenetic changes, supports the survival of PCSCs, which are often induced by stress factors like ROS accumulation, chronic inflammation, and aging. PCSCs are noted for their high self-renewal capacity and altered genome repair ability, resulting in constant mutations and activated redundant self-renewal pathways [157]. These characteristics underscore the importance of the findings that demonstrate the effectiveness of Ps assemblies in targeting PCSCs to combat recurrence in prostate and pancreatic cancers.

The versatility of polymersomes assemblies is also a significant advantage. They offer benefits such as endosomal escape, on-demand cargo release, enhanced intracellular delivery, and low cytotoxicity. Furthermore, the asymmetric architecture of polymersomes, which is composed of a “stealthy” exterior and an internal corona which triggers endosomal escape, exceeds other nanocarriers having symmetrical membranes. This design ensures higher endocytosis rates and improved endosomal escape efficiency.

## 7. Conclusions and Future Perspectives

The future of polymersome-based cancer therapy lies in the exploration of novel materials, stimuli-responsive mechanisms, and targeting strategies to enhance their specificity, efficiency, and safety. Advances in polymer science, bioengineering, and nanotechnology will likely yield more sophisticated polymersome designs with multi-functional capabilities, such as simultaneous drug delivery and diagnostic imaging. Furthermore, integrating polymersomes with emerging therapies, including immunotherapy and gene editing, could open new avenues for personalized cancer treatment.

The future of stimuli-responsive polymersomes in cancer treatment and therapy can register significant advancements, offering promising strategies for overcoming current limitations in cancer care. These nanocarriers, responsive to various stimuli such as pH, temperature, light, hypoxia, and redox conditions, are at the forefront of developing more precise and less invasive treatment modalities. Concretely, some perspectives on their future development can be found below:

(a) Enhanced targeting and specificity: the ongoing research aims to increase the specificity of polymersomes towards cancer cells, while minimizing their impact on healthy tissues. Advanced targeting strategies, which involve the use of tumor-specific ligands or antibodies, are expected to improve selective drug delivery, reducing side effects and enhancing therapeutic outcomes.
(b) Combination therapies: polymersomes offer the unique advantage of codelivering multiple therapeutic agents, including chemotherapeutics, genes, and immunotherapies. Future developments will focus on optimizing these combination therapies to synergistically target cancer cells, overcome drug resistance, and elicit stronger immune responses.
(c) Smart release mechanisms: the development of sophisticated release mechanisms which simultaneously respond to multiple stimuli or in a sequential manner will provide a fine control over drug release kinetics. This could enable the delivery of therapeutics at the optimal time and site of action within the tumor microenvironment.
(d) Personalized medicine: the adaptability of polymersomes makes them ideal candidates for personalized medicine. Future research could focus on designing customized polymersomes to the molecular profile of an individual’s tumor, providing tailored therapies which offer improved efficacy and safety profiles.
(e) Biocompatibility and safety: as the clinical translation of polymersomes advances, ensuring their biocompatibility and safety remains a key aspect. Future studies will need to thoroughly assess the long-term effects of polymersome administration, including their degradation products and the human body’s ability to clear them, in order to meet regulatory standards.
(f) Clinical translation and scalability: efforts to translate polymersomes from the laboratory to the clinic will involve overcoming challenges related to large-scale manufacturing, stability, and storage. Developing cost-effective and scalable production methods will be crucial for making these innovative treatments accessible to a wider population.
(g) Regulatory parties: it is essential to establish clear regulatory pathways for the approval of polymersome-based therapies. Collaboration between researchers, industry, and regulatory agencies will be necessary to bring these novel treatments to market.

## Figures and Tables

**Table 1:** Different methods for preparing polymersomes.

Method	Advantages	Disadvantages	Ref.
Film rehydration	- Simple and straightforward - Suitable for a wide range of amphiphilic copolymers - Large-scale production	- Can result in a mix of vesicle sizes - Potential loss of material - Broad size distribution	[56]
Electroformation	- Produces large, unilamellar vesicles - Good for preparing giant polymersomes - Solvent-free process	- Requires specialized equipment - Limited to certain types of polymers	[57]
Solvent displacement	- Rapid and efficient for polymersome formation - Good for hydrophobic drug encapsulation	- May require postprocessing to remove organic solvents - Can produce a distribution of sizes	[58]
Sonication	- Quick and easy to use - Effective for small-scale preparations	- Can cause heating and degradation of sensitive materials - May produce polydisperse vesicles	[59]
Microfluidics	- Precise control over size and morphology - High reproducibility and scalability	- Requires specialized microfluidic devices - Can be complex to set up and operate	[60]
Self-assembly in selective solvents	- Simple and versatile method - Suitable for a wide range of copolymers	- Solvent choice is critical for successful assembly - May require dialysis or purification steps	[61]

**Table 2:** Overview of postfabrication methods for drug-loaded polymersomes.

Method	Details	Advantages	Disadvantages	Ref.
Extrusion	- Involves passing a polymersome dispersion through membranes with defined pore sizes	- Obtaining uniform-sized polymersomes; - Scalable for larger volumes	- Multiple passes needed for desired size; - Potential shear stress on sensitive molecules	[76]
Electroporation	- Utilizes short electrical pulses to create temporary pores in the polymersome membrane, allowing drugs to enter the vesicles	- Rapid and efficient loading; - Precise control	- Specialized equipment required; - Potential destabilization of polymersomes	[77]
Ultrasonication	- Ultrasound waves are used to agitate the polymersome solution, creating cavitation that disrupts the membrane and allows drug incorporation	- Fast and simple process; - Suitable for heat-sensitive drugs	- Potential degradation of sensitive drugs; - May produce polydisperse vesicles	[78]
Passive loading	- Involves the incorporation of drugs during the self-assembly process of polymersomes	- Simple and straightforward; - Effective for hydrophobic drugs	- Limited control over encapsulation efficiency; - Potential loss of drug during polymersome formation	[79]
Cosolvent evaporation	- Both drug and polymer are dissolved in a common solvent which is then evaporated, leading to drug encapsulation	- Good for encapsulating a wide range of drugs; - Efficient encapsulation process	- Solvent residues might remain; - Potential degradation of sensitive drugs	[71]
Diffusion	- Drugs are diffused into polymersomes from a higher-concentration solution	- Suitable for hydrophilic drugs; - Relatively simple process	- Lower encapsulation efficiency - Potential for drug leakage	[15]

**Table 3:** Works on multi-responsive polymersomes.

Polymersome	Responsiveness	Encapsulated Drug(s)	Targeted Cancer Type	Outcomes	Ref.
mPEG-b-PNIPAM-b-P(DEAEMA-co-BMA)	pH + temperature	DOX and PTX	Breast and cervical cancer	- The release rates of DOX and PTX could be controlled separately; - In vitro efficient uptake of polymersomes by MCF-7 and HeLa cancer cells	[146]
PVCL10-PDMS65-PVCL10		DOX	Cancer therapy	- DOX release under acidic conditions	[147]
UCNP-PNSP@DOX	Ultraviolet + redox	DOX	Non-small-cell lung cancer	- High cell viability against three lung cancer cell lines; - From biochemistry analysis and histopathological results, the nanostructures showed no damage to the heart, liver, spleen, lung, and kidney	[148]
BCP_1–3_Psomes 1.PEG-b-P(FcMA-co-DEAEMA-co-DMIBM) 2.PEG-b-P(FcMA-co-DEAEMA-co-DMIHMA) 3.PEG-b-P(FcMA-co-DEAEMA-co-BPMA)	pH + redox	β-cyclodextrin	Cancer therapy	- High stimuli-responsiveness and drug delivery	[149]
BG-DIP	pH + hyperthermia	ICG and DOX	Breast cancer	- In vivo biodistribution study indicated that BG-DIPs could accumulate in the tumor region, prolong drug retention, and increase photothermal conversion efficiency; - In vivo antitumor study showed that BG-DIPs with laser irradiation efficiently inhibited 4T1-Luc tumor growth with reduced systemic toxicity	[150]
HO-Se-Se(dSe)-PEG-PC7A-P(BAEMA-(AEMA-SS-DOX)	pH + redox	D-peptide antagonist (^D^PPA-1), talabostat, and DOX	Mammary carcinoma	- In vivo results indicated that polymersome could improve tumor accumulation, suppress CAF formation, downregulate regulatory T cells, and promote T lymphocyte infiltration; - In mice, a 60% complete tumor regression ratio and a long-term immune memory response were registered	[151]

## Data Availability

Not applicable.
